# Perceptions of the use of mobile phones to access reproductive health care services in Tamale, Ghana

**DOI:** 10.3389/fpubh.2022.1026393

**Published:** 2022-10-21

**Authors:** John Stephen Agbenyo, Daniel M. Nzengya, Suleiman Kairu Mwangi

**Affiliations:** School of Education and Social Sciences, St. Paul's University, Kiambu, Kenya

**Keywords:** mHealth (mobile health), low- and middle-income countries (LMICs), digital technologies, young people, reproductive health care service

## Abstract

**Introduction:**

Africa has one of the world's highest populations of young people. In addition, Africa has one of the highest proportions of young people facing the worst health challenges. Although previous scholars have reported that young people were using mobile phones to fill in the gaps in accessing reproductive health services, among other health services, there was little comprehensive research on the perception of young people in Tamale, Ghana, on the use of mobile phones to access reproductive health services. This study analyzed the perceptions on mobile phone use to access reproductive health services among young people in Tamale, Ghana.

**Methods:**

The research used a quantitative method design from a target population of 72,706 young people from selected peri-urban, low-income, middle income and high-income residential areas in Tamale Metropolis, Ghana. The sample size used was 397 young people. Participants were selected using a stratified multistage sampling strategy. Descriptive statistics were used to analyse the data.

**Results:**

A total of 86% of the respondents agree that the use of mobile phones helps to overcome cultural challenges that young people in Tamale encounter in accessing reproductive health care. Also, 84.6% of the respondents agree that the use of mobile phones helps them to overcome inadequate access to reproductive health information and services. The use of mobile phones helps to overcome the negative attitude of health providers toward young people in need of reproductive health services was agreed by most of the respondents [strongly agree (35.4%) and agree (49.4%)].

**Conclusion:**

This study informed highly positive perceptions and attitudes toward the use of mobile phones to access Reproductive Health Services in Tamale, Ghana. There is, therefore the need for the health sector to reform its mode of prescriptions of medication, consultation, and service delivery to leverage on the advantages that mHealth presents.

## Introduction

Ghana's population is a youthful population and they can greatly contribute to the socio economic development of Ghana. However, despite the progress in health care delivery, young people are still exposed to health risks which result in premature deaths (1). Young people face various socio-cultural and technological changes that expose them to many health challenges. These challenges limit their choices and can lead to emotional stress, conflict and risk-taking behavior.

In Ghana, social stigma still persists regarding young people's reproductive health issues and health-seeking behaviors (2). Reproductive health is particularly a sensitive issue among young people in Ghana, that many health care providers targeting young people still struggle to address, consequently hindering accessibility to health care services by young people. The health and lives of many young people are jeopardized due to a lack of access to reproductive and sexual health information, services, and supplies, despite the fact that young people play an essential part in fostering the expansion and development of the country. In 2020 alone, the Northern Region of Ghana, of which Tamale, the study area is the capital, recorded 9,249 pregnancies among young girls aged 10–24 (3).

There is a general lack of information regarding sexual and reproductive health (SRH) and this is prevalent among young people (4, 5). There is also a low reported knowledge of reproductive among young people who are in and out of school (6). Young people do not seek health care when they need it because of several barriers, including the difficulty of scheduling appointments, lack of youth-friendly health services, high costs of accessing services, no or limited privacy, confidentiality and the negative and judgemental attitudes of healthcare providers (7).

The use of mobile and wireless technologies (mHealth) can change the way health services are provided around the world and help LMICs reach the UN's Sustainable Development Goals (SDGs) (8). The implementation of the mobile healthcare system in Ghana is slow, and many health services do not deliver the best results (9). Not all young people use mobile phones to access healthcare, and only a small percentage of young people in Ghana use these services after having adopted these services (10). The low patronage of mobile healthcare services raises important questions about why young people fail to seek mHealth care and the factors that influence these decisions, and these studies fill this missing gap.

Sexual activity occurs during one's teenage years. However, young people rarely utilize sexual and reproductive health services and therefore fail to obtain quality treatment. mHealth provides an opportunity for young people to get knowledge on reproductive health. Factors impacting the acceptance of mHealth include users' perception of mHealth platform's ability to resolve healthcare-related issues. Some people believe that mHealth will improve the quality of treatment, enhance connectivity, provide timely information, minimize costs, etc. The perceived benefits of phone-based interventions in improving young people's sexual and reproductive health is high.

Perception influences the use or rejection of new technology. mHealth helps people to increase their self-confidence in the choice of their contraceptive method. Apart from this, mHealth services are noted to give a clearer appreciation of family planning methods or contraceptives (11). Feroz et al. (12) assessed the access to mobile phones between teenage girls and young women in six Nigerian States. They explored the obstacles and limitations to using their phones to seek sexual and reproductive health (SRH) -related information and services. Results of their study showed high access to cell phones but restricted use of mobile phones to access sexual and reproductive health information and services. The perceived obstacles were the shyness in the young women and the justification that young girls are inherently more reserved. This fact is not surprising as the Nigerian community frowns on any attempts made by women to discuss sexual reproductive health issues. As a result, many teenagers in Nigeria are more likely to either be misinformed or poorly informed about SRH issues. The study established a number of barriers, including anonymity, confidentiality, and lack of confidence in service.

Contrary to the above study, which found that mobile phone access was not associated with access to SRH information, studies by Jadhav and Weis (13) found that owning a mobile phone is associated with the overall contraceptive use among people in Uganda, Tanzania, and Haiti. This data was obtained from the Demographic and Health Surveys for six countries by using data collected from 2016 through 2017 for women 15 through 49. The reviewed papers thus reveal that phone access does not completely explain the relationship between SHR uptake and phone access. This depends significantly on how individuals and the various stakeholders understand the role of mobile phones in providing access to reproductive health services. Positive perceptions are likely to influence adoption and vice-versa. Lim et al. (14) show that the low acceptance of using cell phones to seek health information confirms this claim. Study findings found that perceived utility and self-efficacy positively predicted the intention to use a mobile phone for health information. Education is necessary to enable women and men to use mobile phones to access SRHR information and services. The studies by both Jadhav and Weis (13) and Lim et al. (14) focused on respondents outside the UN definition of young people, hence the need for research to address this gap. According to a study by Nuwamanya et al. (15), mobile phone applications in Uganda have the potential to control access to reproductive health information, products, and services. Outcomes of the study were that there was a substantial change in the awareness of SRH, increased contraceptive usage, increased HIV testing, increased STI testing and treatment, and increased condom use.

Similarly, in a study by Gonsalves et al. (16), through the adolescent/youth reproductive mobile access and distribution initiative for love and life (ARMADILLO), young people in Peru and Kenya were better able to dispel contraceptive myths and stereotypes than those who did not have access to ARMADILLO. Based on the findings of the two studies, young people tend to be enthusiastic adopters of digital technology, as evidenced by their ease of using and interaction over multiple platforms on features and smartphones. Additionally, the essence of cell phone technology guarantees a degree of discretion and privacy not available with other contact modes, such as face-to-face communication. However, there is a dearth of evidence about the accessibility of digital health interventions among the rural poor, which leaves a considerable gap in the knowledge.

In Ghana, young people cannot get reproductive health services because of the lack of privacy and poor youth-friendly health services. In response, the Ghana Health Service, which is in charge of the health of the Ghanaian populace, introduced the You Must Know (YMK) mobile application to give young people the privacy and confidentiality they need when accessing reproductive health services and to also attempt to solve the challenge of service availability for young people. From user feedback, it is reported that users thought that the mobile application was easy to find and that it took less time to get to their reproductive health services. They also noted that the YMK app was a simple, flexible, and well-organized system that kept private information private and offered the needed safe space for young people. This perceived ease of use is likely to improve the use of SRH services among YMK users compared to non-YMK users (17). However, no study has explored individuals' perceptions of using mobile phones to access SRH services in the northern region. This study, conducted in Tamale in the Northern Region of Ghana, fills this gap in the literature.

## Materials and methods

The research used a quantitative method design from a target population of young people from selected peri-urban, low-income, middle income and high-income residential areas in Tamale Metropolis in Ghana. Tamale is the regional capital of the Northern region of Ghana. It is one of the 26 districts in the Northern Region of Ghana and is reported to be the third largest city in Ghana, after Accra and Kumasi. According to the 2014–2017 Medium Term Development Plan of the Tamale Metropolitan Assembly, there are a total of 116 communities, 41 of which are considered to be urban communities, 17 of which are considered to be peri-urban, and 58 of which are considered to be rural in nature. Tamale has been selected for this research because, according to the statistical service, Tamale is the fastest growing metropolitan area after Greater Kumasi (18). According to the 2021 Population and Housing Census, the city is the fourth largest city in Ghana, with an estimated population of 374,744, with 185,051 males and 189,693 females (19). Tamale is one of the five predominant areas with urban population living in slums (20). Tamale is listed among the fastest growing cities in West Africa, with the majority of the residents being Muslims, and they practice the polygamous marriage system with large family sizes (21). The city's population is young, with almost 36.4% of the population under the age of 15 (22). Tamale has attracted immigrants from poor rural areas in northern Ghana and has been growing at an average of 4% per year for the past ten years.

The sample size of 397 was determined based on the Krejcie and Morgan's (23), sample size calculation and determination based in the population of 72,706. Young people aged 10–24 years located within six (6) selected communities (Vittin- Target, Tutigli, Kalariga, Tishigu, Warizehi and Lamashegu) in Tamale, Ghana were interviewed. Participants were selected using stratified multistage sampling strategy. A three-stage sampling procedure was used to select the study respondents. A sample of primary units was chosen, a secondary unit sample was picked from each of the selected primary units, and a tertiary unit sample was selected from the secondary units.

The study used a standardized questionnaire that provided quantitative information. To measure the perception of young people, regarding the use of the mobile phone to access reproductive health services, thirteen (13) test items assessing different aspects including how mobile phones can be used to manage reproductive health, capability to increase reproductive health management, overcoming cultural challenges using mobile phones, bridging the inadequacy of reproductive health information using mobile phone among other aspects of individual perceptions. Three of the test items were on a 3-point scale (*Agree, Neutral and Disagree*) while 10 test items were on a 2-point scale (Agree, Disagree). Descriptive statistics were used to analyse the data on perceptions of young people about the use of mobile phones to access reproductive health services.

To ensure proposer data management, as soon as the data collected from respondents were entered into SPSS, the paper forms were stored in a locked cabinet at the private residence of the Principal investigator, and this was accessible only to the Principal Investigator. Upon the completion of the research project, all paper forms were destroyed through shredding.

## Results

This section presents the results on the perceptions of young people on using mobile phones to access reproductive health services in Tamale. The findings of this research revealed that more than half (52.9%) of the respondents agreed that the use of mobile phones to access reproductive health services, helps them to manage their daily reproductive healthcare needs more quickly. Only a few, (21%) of the respondents disagreed that using the mobile phone to access reproductive health services helped them in managing their daily reproductive healthcare needs more quickly. Slightly more than those who disagreed (26.1%) indicated that they were neutral on whether using a mobile phone to access reproductive health services helps them manage their daily reproductive healthcare needs more quickly.

From the survey findings, a majority (61%) of the participants who took part in the study agreed that using the mobile phone to access reproductive health services increases their capability to manage their reproductive health. A small number of the respondents (13.7%) disagreed that using the mobile phone to access reproductive health services increases their capability to manage their reproductive health. There was a reasonable number of 25.3% of the respondents who opted to be neutral on whether the use of mobile phones to access reproductive health increases their capacity to manage their reproductive health or not.

This study further found that a majority (69%) of the survey respondents agreed that using a mobile device to search for reproductive health information was beneficial to them. A significant number of the survey respondents (24.1%) were neutral on whether using a mobile device to search for reproductive health information was beneficial to them or not. A very small percentage of respondents (6.9%) of the survey respondents disagreed with the assertion that using a mobile device to search for reproductive health information was beneficial to them.

Using mobile phones to access reproductive health services helps young people to overcome cultural challenges that young people in Tamale encounter. This was agreed upon by almost all respondents (85.8%) of the survey. Only a small number of the survey respondents (14.2%) disagreed with the assertion that the use of mobile phones helps them to overcome cultural challenges that young people in Tamale encounter in accessing reproductive health care.

A majority of the study respondents (84.6%) agreed that the use of mobile phones helps to overcome the lack of access to reproductive health information and services in the Tamale metropolis. Only a small number (15.4%) were not in agreement that the use of mobile phones helps to overcome inadequate access to reproductive health information and services. Also, 84.8% of the survey respondents reported that the use of mobile phones helps to overcome the negative attitude of health providers toward young people in need of reproductive health services. Only 15.2% of the respondents disagreed that mobile phones helped them overcome the negative attitude of health providers toward young people who needed reproductive health services.

This research found that a majority (82.5%) of the study respondents agreed that the use of mobile phones to seek reproductive health services helps them to overcome the lack of information on reproductive health service points. Only 17.5% of respondents were not in agreement that the use of mobile phones helped them overcome the lack of information on reproductive health service points.

The research further sought to find out from the survey respondents whether the use of mobile phones to access reproductive health services helps them to overcome the impact of religious teachings on the access to reproductive health services by young people in Tamale. More than half (79.5%) of the study respondents agreed that using mobile phones helps overcome the impact of religious teachings on the access to reproductive health services by young people in Tamale. Even though the majority of survey respondents were in agreement that the use of mobile phones helps to overcome the impact of religious teachings against the access to reproductive health services by young people in Tamale, 20.5% of the survey respondents disagreed that the use of mobile phones helps them to overcome the impact of religious teachings against the access to reproductive health services by young people in Tamale.

The study found that 81.3% of the study respondents, representing a majority of the sampled young people, agreed that using mobile phones to access reproductive health care information and services is an effective way to overcome the limited reproductive health care information and service delivery outlets. Among the respondents, 18.7% disagreed with the majority's opinion that, the use of mobile phones can help overcome the limited reproductive health care information and service delivery outlets.

This study further revealed that a majority of the study respondents (86.3%) agreed that using mobile phones makes it easier for young people to access reproductive health information and services for much less money. Only 13.7% of the respondents disagreed that using mobile phones helped them to overcome the challenge of the high cost of assessing reproductive health information and services. Similarly, 86.1% of the study respondents agreed that the use of mobile phones to access reproductive health services, helps in overcoming the challenge associated with distance to health facilities. However, 13.9% of the study participants disagreed that using their mobile phones to access reproductive health services helped them to overcome the challenge associated with distance to health facilities.

A majority (84.3%) of the study respondents concurred that using mobile phones helped them overcome the challenge of not knowing the accuracy of the information young people receive from their friends. Only 15.7% of the study participants disagreed with this.

Finally, a majority (89.1%) of the study respondents agreed that using mobile phones helps them overcome the fear of being judged and stigmatized as a result of assessing in-person reproductive health services. Only a small percentage of 10.9, disagreed that using mobile phones helps them overcome the fear of being judged and stigmatized as a result of assessing reproductive health services, as shown in Table 1 of this report.

**Table 1:** Perceptions about the use of mHealth.

**Variable**	**Category**	**%**
Using the mobile phone to access reproductive health services helps me in managing my daily reproductive healthcare needs more quickly	Disagree	21.0%
	Neutral	26.1%
	Agree	52.9%
Using the mobile phone to access reproductive health services increases my capability to manage my reproductive health	Disagree	13.7%
	Neutral	25.3%
	Agree	61.0%
Using my mobile device to search for reproductive health information is beneficial to me.	Disagree	6.9%
	Neutral	24.1%
	Agree	69.0%
Level of agreement/disagreement to the following statements		
The use of mobile phones helps to overcome cultural challenges that young people in Tamale encounter in accessing reproductive health care	Agree	85.8%
	Disagree	14.2%
The use of mobile phones helps to overcome inadequate access to reproductive health information and services	Agree	84.6%
	Disagree	15.4%
The use of mobile phones helps to overcome negative attitude of health providers toward young people in need of reproductive health services	Agree	84.8%
	Disagree	15.2%
The use of mobile phones helps to overcome the lack of information on reproductive health service points	Agree	82.5%
	Disagree	17.5%
use of mobile phones helps to overcome the impact of religious teachings against the access of reproductive health services by young people in Tamale	Agree	79.5%
	Disagree	20.5%
The use of mobile phones helps to overcome the limited reproductive health care information and service delivery outlets	Agree	81.3%
	Disagree	18.7%
The use of mobile phones helps to overcome the challenge of high cost of assessing reproductive health information and services	Agree	86.3%
	Disagree	13.7%
The use of mobile phones helps to overcome the challenge associated with distance to health facilities	Agree	86.1%
	Disagree	13.9%
use of mobile phones helps to overcome the challenge of Not knowing the accuracy of the information young people receive from their friends	Agree	84.3%
	Disagree	15.7%
The use of mobile phones helps to overcome the fear of being judged and stigmatized as a result of assessing reproductive health services	Agree	89.1%
	Disagree	10.9%

## Discussions

Perception influences the use or rejection of new technology. mHealth helps people to increase their self-confidence in the choice of their contraceptive method and is noted to give a clearer appreciation of family planning methods or contraceptives (11). This study found a high perception of mHealth use. The findings of this research, which showed that more than half (52.9%) of the respondents agreed that the use of mobile phones to access reproductive health services helped them to manage their daily reproductive healthcare needs more quickly, indicating a higher level of agreement than the findings of Macharia et al., (24), who reported that 21.6% (29/134) of their respondents reported improved decision-making as a result of using mHealth services. Hill et al., (25), report that mHealth services are used to change behavior and to increase the uptake of reproductive health services. Other research evidence suggests that mHealth interventions are an effective way to increase respondents' autonomy to make the needed decisions on their preferred contraceptive methods because, through their mobile phones, they have unlimited access to readily available, complete, and validated information (11).

A person's ability to make sound reproductive health decisions has been known to be strongly impacted by the person's level of knowledge and awareness. Dev et al. (26), found that people who understood family planning were more inclined to use it. People's ability to make decisions and empower themselves has also been given a boost through the use of mobile interventions. People's knowledge, attitudes, and practices regarding modern contraception may be influenced by the availability and use of mobile health solutions, which helps them better understand family planning alternatives. Smith et al. (27), reported that their study participants had difficulties retaining information on their family planning methods. However, they reported that mHealth services helped them to retain useful information about family planning contraception, which was difficult before the mHealth intervention.

This study found that a majority (69%) of the survey respondents agreed that using a mobile device to search for reproductive health information was beneficial to them. This suggests a high positive perception of respondents for the use of mHealth. Although the findings suggest that more than half of the respondents have a positive perception of the use of mHealth, significant numbers show neutrality in their responses. A probable explanation for this could be that they may not have had any encounter yet with an mHealth service and thus, are not able to explicitly state a position on the use of mHealth. Using mobile phones to access reproductive health services can increase decision-making and empower users of mHealth services (26). mHealth is more advantageous than face-to-face contact with health care providers (28). The advantages of mHealth can be viewed from privacy, where the person accessing the information does not necessarily need to have a face-to-face encounter with anyone, stress-free because one can sit in the comfort of one home or anywhere and get the needed information. From the economic perspective, because the message is by SMS or the fact that one does not need to travel to a health center, it saves money and time, which are invaluable.

Using mobile phones to access reproductive health services helps young people to overcome cultural challenges that young people in Tamale encounter. This was agreed upon by almost all respondents (85.8%) of this research. The Ghanaian culture considers discussions about sexuality as a sacred topic with young people, and thus, discussions about sexuality are perceived generally as introducing young people to early sexual intercourse. Among some ethnic groups in Ghana, it is considered an abomination to talk about sexual issues with a child because the belief is that the child could be 'spoilt'. This further goes to the point where even if the child needed to find out certain things about sexuality, the child was told he or she was not of age to know about such issues (29). The socio-cultural factors such as stigma, myths and misconceptions are reported to have negatively affected the provision of reproductive health services and have hindered the delivery and utilization of sexual reproductive health services for young people (30). These findings, therefore, suggest that young people can leverage on mHealth services to access all of their reproductive health needs without the fear of being subjected to the cultural barriers that they would ordinarily have faced.

A majority of the study respondents (84.6%) agreed that the use of mobile phones helps them to overcome the lack of access to reproductive health information and services in the Tamale metropolis. This suggests that young people have a strong positive perception about the use of mobile phones to access reproductive services and bridges the barrier of poor health worker attitude toward them. This finding supports the earlier finding of Feroz et al. (28), in their study to understand how the use of mobile phones can improve the uptake of antenatal and post-natal services in Pakistan. Feroz et al. (28), reported that a large number of their respondents were willing to use mHealth because they considered it to be more beneficial than the face-to-face communication that they had previously experienced with health care workers.

mHealth solves many of the problems that young people face when they are looking for reproductive health information and services. When young people try to get reproductive health information and services, they often say that health care workers make them feel bad about themselves or treat them badly. Young people can access any sexual and reproductive health information and service if youth-friendly services are provided. Research has demonstrated that young people place high importance on maintaining their privacy while obtaining sexual and reproductive health information and services, and these same young people are more likely to seek sexual and reproductive health treatment and interventions when their confidentiality is ensured (31). Additionally, young people, both married and unmarried, are usually disregarded, and there is a general lack of youth-friendly services. The attitude of providers is frequently cited as one of the most significant obstacles to accessing health care in a variety of LMIC settings. Many health workers discourage young people from using services because of a lack of confidentiality, attitudes of judgement, disdain, or a failure to take seriously, the sexual and reproductive health needs of adolescents. (32). The cost and getting to medical facilities can also be a problem. Young people find it hard to get good, comprehensive information about sexual and reproductive health because they feel embarrassed and think adults do not care about their privacy and confidentiality (33).

Consistent with earlier research, this research found that a majority (82.5%) of the study respondents agreed that the use of mobile phones to seek reproductive health services helps them to overcome the lack of information on reproductive health service points. This suggest that mHealth fills in the gap of the lack of information on youth-friendly service points. This is consistent with the works of Abrejo et al. (11), who reported that mHealth fills in the gap in information around family planning methods. Sexual and reproductive health information sent through mobile phones have the potential to raise awareness rapidly and to spread to a larger audience than was originally intended. It has been observed that the use of mobile phones, in particular smartphones, might satisfy the curiosity of young people regarding the changes that are taking place in their bodies as well as the desire that young people have to gain knowledge of new things. Through the use of mobile phones, young people would have access to helpful information and be able to clear up any misunderstandings (8).

The research sought to find out from the survey respondents whether the use of mobile phones to access reproductive health services helps them to overcome the impact of religious teachings on the access to reproductive health services by young people in Tamale. More than half (79.5%) of the study respondents agreed that using mobile phones helps overcome the impact of religious teachings against the access to reproductive health services by young people in Tamale. This suggests that young people have a positive perception of the use of mHealth as they believe that it helps them to overcome the impact of religious teachings against access to reproductive health services.

81.3% of the study respondents are in agreement that using mobile phones to access reproductive health care information and services is an effective way to overcome the limited reproductive health care information and service delivery outlets. This suggests that, for communities that have few health facilities as well as few service delivery outlets, mHealth can bridge these gaps. The finding of this research is consistent with earlier research by Ochieng et al. (8), who concluded that mobile phones could help adolescents learn and access helpful information about sexually transmitted infections (STIs) and how to avoid them. The study further found that 86.3% of the respondents agree that using mobile phones makes it easier for people to access reproductive health information and services for much less money. This is consistent with the study by Abrejo et al. (11), who found that respondents believed that mobile phones would save them the cost of transportation they would usually have had to incur if they wanted to get information about their reproductive health or receive services related to it at a health facility.

The accuracy of information is a crucial factor in determining young people's choice in the use of mHealth services, especially today, where information seems to abound. A majority of the study respondents concurred that using mobile phones helps them overcome the challenge of not knowing the accuracy of the information young people receive from their friends. The findings from this research suggest that with mHealth, young people trust that they will have access to up-to-date and accurate information, regarding their sexual and reproductive health, as this will assist them in making more informed decisions that will be beneficial to their overall health.

The response from the high proportion of respondents (89.1%) who agree that the use of mobile phones helps to overcome the fear of being judged and stigmatized as a result of assessing reproductive health services is consistent with similar studies from Alhassan et al. (34), who found in their studies that the use of mobile phones provides young people with the anonymity as well as confidentiality, and privacy that they need, and this helps to reduce the discrimination, stigma, and the shyness that is associated with young people's access to STIs services at various health facilities.

## Conclusion and recommendations

The findings of this study on the perceptions of young people on the use of mHealth show that young people have a positive perception of using mobile phones to access reproductive health services. These findings are consistent with those of Obasola and Mabawonku (35), who reported that women had positive perception of sexual and reproductive health interventions that are ICT-based. They analyzed that mobile health interventions were appealing to young people. Young people positively perceive health-related apps because they are convenient and safe and offer the utmost privacy. The results show that using mobile phones for reproductive health services provides quick access to information and services.

The high positive perception rates recorded confirm that there is value in using mobile phones to access reproductive health services as it addresses a number of critical barriers that young people face in assessing reproductive health services. Mobile phones have been proven to be a safe, cost-effective, and reliable method of delivering acceptable, secure, and accurate information and services on sexual and reproductive health (36). Using mobile phones for reproductive health care can improve health awareness and is generally well-received by young people (37). There are several hurdles that young people encounter in their quest to access reproductive health and information and services. These, which include privacy and secrecy in accessing sensitive materials and information, were reported to be resolved by the advent of mHealth and the use of mobile phones (30, 38). This current study provides evidence that using mobile phones helps young people overcome the fear of being judged and stigmatized as a result of assessing reproductive health services. Young people place a high premium on maintaining complete secrecy whenever they access sexual reproductive health services. (24). When young people seek information and services related to their sexual and reproductive health, they want to be assured that their confidentiality will be respected and safeguarded.

Health care providers and policymakers must strive to implement multi-faceted patient engagement strategies to provide young people with the resources to manage their reproductive health needs on their own, as innovations such as mHealth continue to permeate the industry.

Service providers and policymakers should explore incorporating technology in addressing the challenges faced in accessing health care in Ghana. The use of mobile phon33e technology can also be applied to medical consultations that are traditionally done in consulting rooms in health facilities. The health facilities can institute the mHealth applications to help remove some barriers young people face in accessing reproductive health services.

## Data availability statement

The original contributions presented in the study are included in the article/supplementary material, further inquiries can be directed to the corresponding author.

## Ethics statement

The studies involving human participants were reviewed and approved by Navrongo Health Research Center Institutional Review Board (NHRCIRB), Ghana. Written informed consent to participate in this study was provided by the participants' legal guardian/next of kin.

## Author contributions

JA led in the conduct of the research by collecting data, analyzing the data, and writing the draft manuscript. DN and SM provided overall supervision of the research by supervising the data collection and data analysis and edited the manuscript. All authors read, contributed to the research design, and approved the final manuscript.

## Conflict of interest

The authors declare that the research was conducted in the absence of any commercial or financial relationships that could be construed as a potential conflict of interest.

## Publisher's note

All claims expressed in this article are solely those of the authors and do not necessarily represent those of their affiliated organizations, or those of the publisher, the editors and the reviewers. Any product that may be evaluated in this article, or claim that may be made by its manufacturer, is not guaranteed or endorsed by the publisher.
